# Comment on “A Sustained Reduction in Serum Cholinesterase Enzyme Activity Predicts Patient Outcome following Sepsis”

**DOI:** 10.1155/2019/5012874

**Published:** 2019-04-23

**Authors:** Carlo Chiarla, Ivo Giovannini

**Affiliations:** CNR-IASI Center for the Pathophysiology of Shock and Biomathematics, Catholic University of the Sacred Heart School of Medicine, Rome, Italy

We consider extremely interesting the article on the prognostic implications of reduced serum butyrylcholinesterase activity (BChE-A) in sepsis by Zivkovic et al., which was published in [1]. Moreover, this group of investigators previously characterized the decrease in BChE-A in severe systemic inflammation in critical illness and brought other important contributions to the topic in this same journal [2] and elsewhere. Their work, by using a point-of-care testing system for frequent BChE-A monitoring, could provide very refined information.

We would like to comment on two aspects, the first and minor one being the establishment of precise cut-off values of BChE-A for the risk of poor outcome. In fact, there are different methods in different hospitals to assess BChE-A, with changes in the range of normality. This range is not even indicated in some articles, and sometimes values smaller than the lower normal limit are not exactly reported. Of course, some “translation” of the observed BChE-A from a given range to the different range of another method is feasible; however, it is with very approximate results. The establishment of precise cut-offs by statistical means [1] is a crucial step to progressively improve risk stratifications; however, by now, the key general finding (we believe) remains the clinical relevance of very low BChE-A as an index of poor prognosis, an issue that many physicians are not yet familiar with. Perhaps there is also a need to unify methodologies of BChE-A determination. In our patients, BChE-A nearing or below 1/4 the lower normal limit (we cannot be more precise because of the various methodologies used over the years) has always been among the alarming signs which imposed the strictest attention and care.

A major issue, which more often regards surgical patients, is the fact that BChE-A is acutely decreased by the loss of ascitic and pleural fluid (through paracentesis, thoracentesis, and surgical drainages) or bleeding and is acutely increased by transfusion of fresh frozen plasma, even independently of severity of illness. Our familiarity with these patterns developed over some decades of observation. We became used to witness the association of decreasing BChE-A with surgical trauma, complications like sepsis and poor prognosis, and the association of slowly increasing BChE-A with clinical improvement, but evident exceptions to these “customary” changes were falls in BChE-A after recent loss of ascitic and pleural fluid or some bleeding and increases in BChE-A after transfusion of plasma. Ignoring such events when assessing BChE-A at a single time point (i.e., upon admission to the intensive care unit) acts as a confounding factor. At least, to our knowledge, the issue is ignored in most publications, although Zivkovic et al. marginally mentioned it in this journal [2] and we partly did so elsewhere [3]. Precise quantification of these effects may not be easy. It depends on volumes of lost fluids and blood, of transfused plasma, on BChE-A in these volumes (which varies even in ascitic and pleural fluid) [4, 5] and patterns of redistribution. Our practical approach is just to avoid straightforward inclusions of changes in BChE-A among the signs of a worsening or an improving condition after those events. As an extreme example, in a patient with a combination of liver dysfunction and sepsis, BChE-A increased from 1909 to 3950 U/L (n.v. 3900-11500) after plasmapheresis, falling back near the starting value in 3 days in the absence of clinical improvement.

This point may also regard the unknown mechanisms linking low BChE-A to poor prognosis. BChE-A may be thought of also in terms of weight concentration [6] or body pool of enzyme protein, and insufficiency of this pool does not seem to be an explanation. In sepsis, the infusion of fresh frozen plasma does not cause but a very transient increase in BChE-A, without clinical improvement unless the underlying condition is resolved.

Finally, in our database of surgical and critically ill patients, in which we found relatively tight covariation of BChE-A with albumin and cholesterol (characterizing these three components as “negative” components of the acute phase response), the following unpublished regression showed that in the presence of sepsis BChE-A underwent a greater fall for any given albumin and cholesterol level:

(1) $$\begin{array}{c} \text{BChE}‐\text{A}=\left[307.97\cdot 1.07^{\text{albumin}}\cdot 1.06^{\text{cholesterol}}\right]\cdot 0.83_{\text{SEPSIS}} \end{array}$$

*n* = 421; *r*^2^ = 0.65; *p* < 0.001 for all coefficients and whole regression:

BChE-A, U/L, n.v. 5300-13000; albumin g/L; cholesterol mmol/L. The last coefficient (0.83_SEPSIS_) accounts for the mean fall in BChE-A associated with the presence of sepsis, for any given albumin and cholesterol level.

This suggests that BChE-A in sepsis might be a more sensible marker of risk, in addition to being a potentially important biochemical monitor of extrasynaptic cholinergic activity [1, 7].

In conclusion, the finding of reduced serum BChE-A in “infection” can be traced back at least to about 80 years ago [8] and it is frustrating that we are still unaware of all the involved aspects, although the article of the authors [1] surely stands as one of the best available and useful sources of information on BChE-A in sepsis and critical illness.
